# Magnetic Microrobots Fabricated by Photopolymerization and Assembly

**DOI:** 10.34133/cbsystems.0060

**Published:** 2023-11-14

**Authors:** Xiyue Liang, Yue Zhao, Dan Liu, Yan Deng, Tatsuo Arai, Masaru Kojima, Xiaoming Liu

**Affiliations:** ^1^ Key Laboratory of Biomimetic Robots and Systems, Ministry of Education, State Key Laboratory of Intelligent Control and Decision of Complex System, Beijing Advanced Innovation Center for Intelligent Robots and Systems, and School of Mechatronical Engineering, Beijing Institute of Technology, Beijing 100081, China.; ^2^ Center for Neuroscience and Biomedical Engineering, The University of Electro-Communications, Tokyo 182-8585, Japan.; ^3^ Department of Materials Engineering Science, Osaka University, Osaka 560-8531, Japan.

## Abstract

Magnetic soft microrobots have great potential to access narrow spaces and conduct multiple tasks in the biomedical field. Until now, drug delivery, microsurgery, disease diagnosis, and dredging the blocked blood vessel have been realized by magnetic soft microrobots in vivo or in vitro. However, as the tasks become more and more complex, more functional units have been embedded in the body of the developed magnetic microrobots. These magnetic soft microrobots with complex designed geometries, mechanisms, and magnetic orientation are now greatly challenging the fabrication of the magnetic microrobots. In this paper, we propose a new method combining photopolymerization and assembly for the fabrication of magnetic soft microrobots. Utilizing the micro-hand assembly system, magnetic modules with different shapes and materials are firstly arrayed with precise position and orientation control. Then, the developed photopolymerization system is employed to fix and link these modules with soft materials. Based on the proposed fabrication method, 3 kinds of soft magnetic microrobots were fabricated, and the fundamental locomotion was presented. We believe that the presented fabrication strategy could help accelerate the clinical application of magnetic microrobots.

## Introduction

Untethered microrobots are showing great potential in biomedical fields. Among the existing untethered microrobots, magnetically controlled soft microrobots demonstrate the advantages of fast response, strong actuation, programmable deformability, and multiple functions [1–6]. Until now, drug delivery, microsurgery, disease diagnosis, and dredging the blocked blood vessel have been realized by magnetic microrobots in vivo or in vitro [7–11]. However, as the tasks become more and more complex, more functional units are embedded in the body of the developed magnetic microrobots. These magnetic soft microrobots with complex designed geometries, mechanisms, and magnetic orientation are now greatly challenging the fabrication of the magnetic microrobots [12].

In the fabrication of magnetic soft microrobots, the 2-dimensional (2D)/3-dimensional (3D) printing methods and casting with the printed mold are the most commonly adopted strategies [13–17]. Among the existing 3D printing methods, photopolymerization holds the advantages of high fabrication speed and outstanding accuracy [18–25]. Typically, photopolymerization can directly fabricate the magnetic soft microrobots by polymerizing particular local areas in liquid photopolymer containing magnetic particles or other magnetic materials by light in a layer-by-layer manner [14,26–32]. Then, the magnetic soft microrobots are functionalized by magnetization. Nowadays, to meet the requirement of programmable deformation, magnetization is conducted with local polymerization at the same time. However, the interaction between the neighbor magnetic particles in the unpolymerized liquid photopolymer caused by the magnetic field for magnetization will influence the programmed magnetization [33]. Moreover, photopolymerization restricts fabricating the magnetic soft microrobots with various photopolymer materials [34]. The above challenges in fabricating magnetic soft microrobots by photopolymerization strictly limit realizing the promising designs, miniaturizing, and enriching the functions.

Micro-assembly promoted by the developing micromanipulation technology could now fabricate micro-electric and mechanical machines with multiple components or even artificial tissues with basic modules [35–38]. Fabricating the magnetic soft microrobots by assembling the micro-magnetic modules through precisely positioning the modules and controlling the orientation could flexibly select various materials and form strong programmed local magnetic torque vectors. Assisted by computer vision under the microscope, realized robotic micro-assembly is more efficient [39–42]. However, the time used to fabricate a microrobot with the same size and same accuracy by micro-assembly is often more than 10 times that of photopolymerization. Thus, a promising solution is combining photopolymerization and micro-assembly in the fabrication of the magnetic soft microrobots, which is aligning the magnetic micro modules by micro-assembly and linking these modules by photopolymerization to form the body finally [43–47].

In this paper, we propose a new method combining photopolymerization and micro-assembly for the fabrication of magnetic soft microrobots. Utilizing the micro-hand assembly system, magnetic modules with different shapes and materials were firstly arrayed with precise position and orientation control. Then, the developed photopolymerization system was employed to fix and link these modules with soft materials. Based on the proposed fabrication method, 3 kinds of soft magnetic microrobots were fabricated, and the fundamental locomotion was presented. The remaining parts of this article are organized as follows: Firstly, the overall fabrication principle and system and the detailed fabrication process are introduced. Then, the test results of the fabricated three magnetic soft microrobots are presented. Finally, we discuss the advantages of the proposed fabrication method for magnetic soft microrobots.

## Materials and Methods

**Fig. 1.**
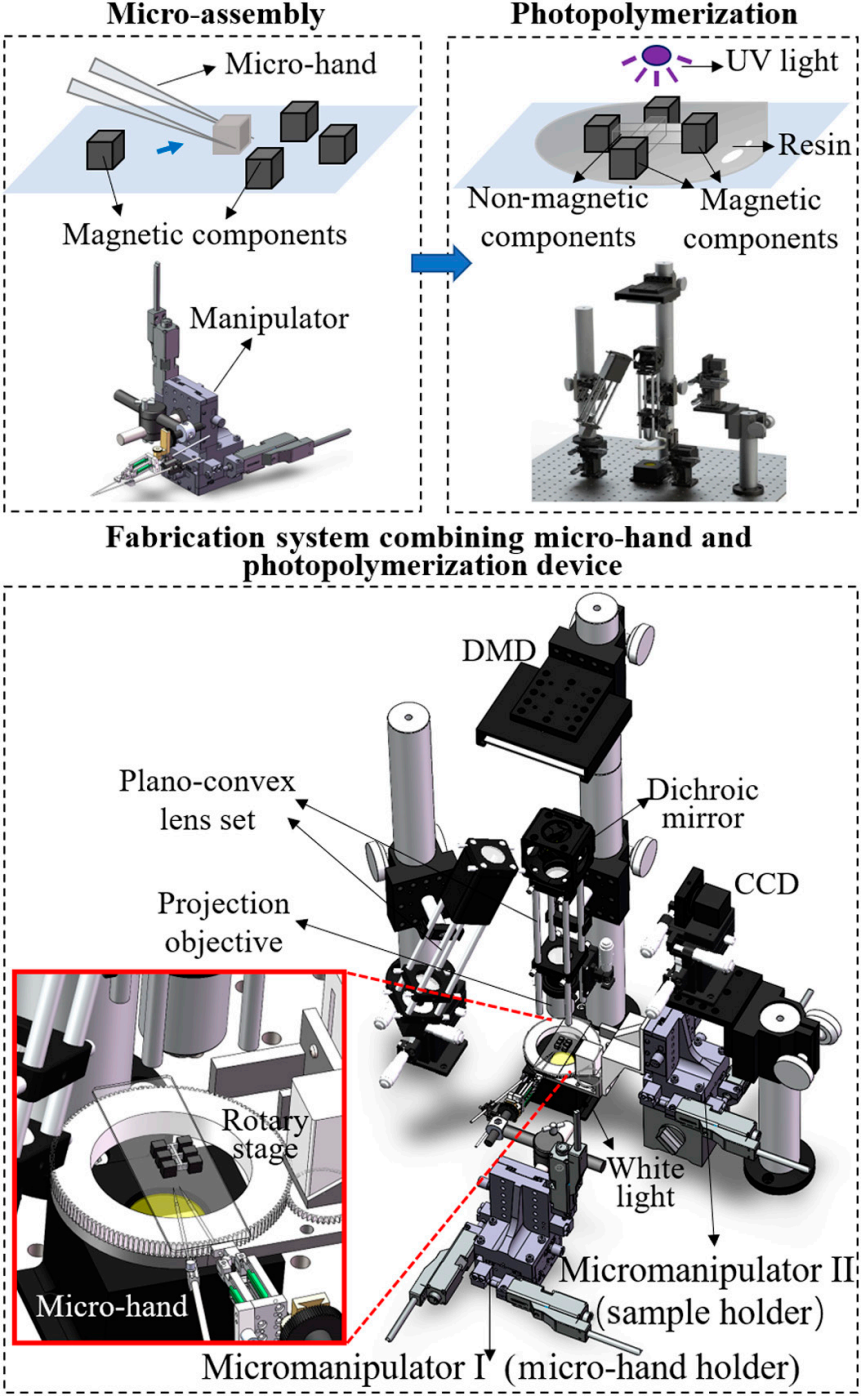
Fabrication system combining the micro-hand and the photopolymerization device.

As shown in Fig. 1, the whole fabrication system combined the micro-assembly and the photopolymerization devices. The micro-assembly system was utilized to position the pre-magnetized micro modules and control their orientation. Then, the photopolymerization device was employed to form the soft body of the magnetic microrobot and package the aligned magnetic micro modules inside.

The micro-assembly was mainly conducted by a piezo-driven 2-finger micro-hand and a rotary stage mounted on two three-degree-of-freedom (3-DoF) micromanipulators separately. The 2-finger micro-hand was responsible for grasping the well-oriented pre-magnetized micro modules and releasing them at the desired position. The Micromanipulator I (Micro-hand holder) could hold the whole micro-hand and move in a big 3D workspace, which enabled assembling the magnetic module array from microscale to macroscale. The rotary stage was utilized to rotate the micro magnetic module to achieve a proper posture for grasping. The assembled array on the substrate should also be positioned and rotated to make sure the released micro magnetic module could achieve not only the designed position but also the desired orientation. These were all achieved by the Micromanipulator II (Sample holder) with the rotary stage mounted on it.

The micro-hand was a 3-DoF parallel mechanism driven by 3 piezoelectric actuators. The motion of every joint was realized through the designed flexure hinge. The piezoelectric actuator (NEC TOKIN, AE0203D16) with a maximum extension of 17 μm was driven by a voltage amplifier (Matsusada Precision Inc., PZJ-0.15P). Two strain gauges were orthogonally attached to the piezoelectric actuator to realize the close-loop control and avoid hysteresis. The feedback signal was conditioned by the strain gauge bridge box and amplified by the amplifier (Kyowa, MCD-8A). The signal was then input into the micro-hand controller (AD chip ADC78H90, Da chip DAC-AD5363, MCU STM32 F767ZI) for Analog-to-Digital (AD) conversion. The onboard motion control algorithm of both the micro-hand and micromanipulators was executed on this controller. The Micromanipulator I consisted of an X-Y-Z stage (OptoSigma, TAM 655) and 3 linear motors (OptoSigma, SGSP-13ACT-B0) with a maximum travel distance of 13 mm and a maximum speed of 2 mm/s.

The rotary stage was mounted on Micromanipulator II with the same configuration as Micromanipulator I. The rotary stage was printed by a 3D printer (Stratasys, uPrint SE). The rotary stage consisted of a bearing (NB 6810ZZ, NSK) with an inner diameter of 50 mm, an outer diameter of 65 mm, a thickness of 7 mm, and a rotational internal clearance from 1 to 15 μm. The snap ring groove was placed on a cap-like substrate. A circular groove with gear tooth outside covered the snapping ring. The circular groove cover is rotated by a 5-phase stepper motor (Oriental Motor, PK513PA) with a small gear.

The photopolymerization sub-system was composed of the digital micromirror device (DMD), a dichroic mirror (Daheng Optics, GCC-424001), a plano-convex lens set (Daheng Optics, focal length 50 mm, spacing 100 mm), a projection objective (Daheng Optics, 10×), and a white light set (Daheng Optics, GCC-300115). The ultraviolet (UV) light source came from the tilted plano-convex lens set on the left. The image was captured by a charge-coupled-device (CCD) camera (HIKROBOT, MV-CA050-11UM) with a resolution of 2,448 × 2,048 and a frame rate of 35 frames/s. The images were processed by computer vision and provided position and posture feedback to the micro-hand controller. The photopolymerization procedure was also evaluated by computer vision, and the decision was made and sent to the DMD.

**Fig. 2.**
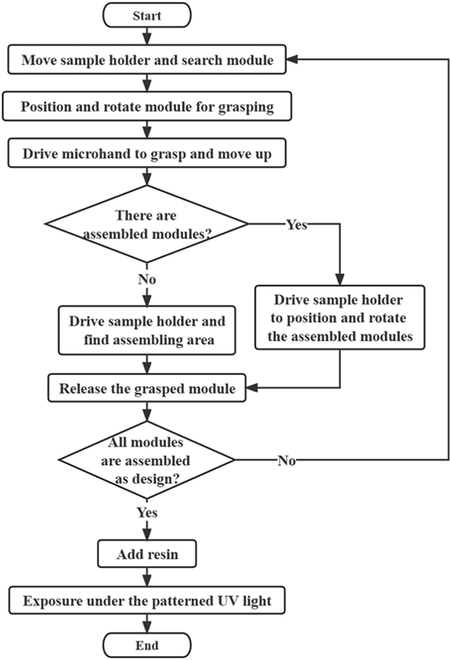
Detailed fabrication process according to the proposed fabrication method.

As shown in Fig. 2, we designed the detailed fabrication process according to the proposed fabrication method. Firstly, we conduct the grasping process. We moved the sample holder and searched for pre-magnetized micro modules in the field of view. After finding the proper module, it would be moved to the visual field center and then rotated to achieve a proper orientation for grasping by the rotary stage. Then, we drove the 2-finger micro-hand to grasp the module and move it up by the micro-hand holder. Secondly, the system started the release process. The sample holder moved the substrate to find the assembly area where the grasped modules should be placed. If assembled modules were in the assembling area, the assembled modules should be positioned and oriented. Then, it is ready to accept the released modules held by the micro-hand located upon the center of the visual field. Now, we can move the grasped micro module down by the micro-hand holder and release it on the substrate by opening the micro-hand. Following the above process, the micro modules could be assembled individually with precise position and orientation control. At last, after all pre-magnetized micro modules were assembled as designed, the resin was added until the resin covered the whole assembling area. Then, the UV light source was turned on and patterned by the DMD. The liquid photopolymer at the local exposure area is photopolymerized according to the designed pattern. The pre-magnetized micro modules were packaged in the photopolymerized soft body, and the fabrication of the target magnetic soft microrobot was finally completed.

**Fig. 3.**
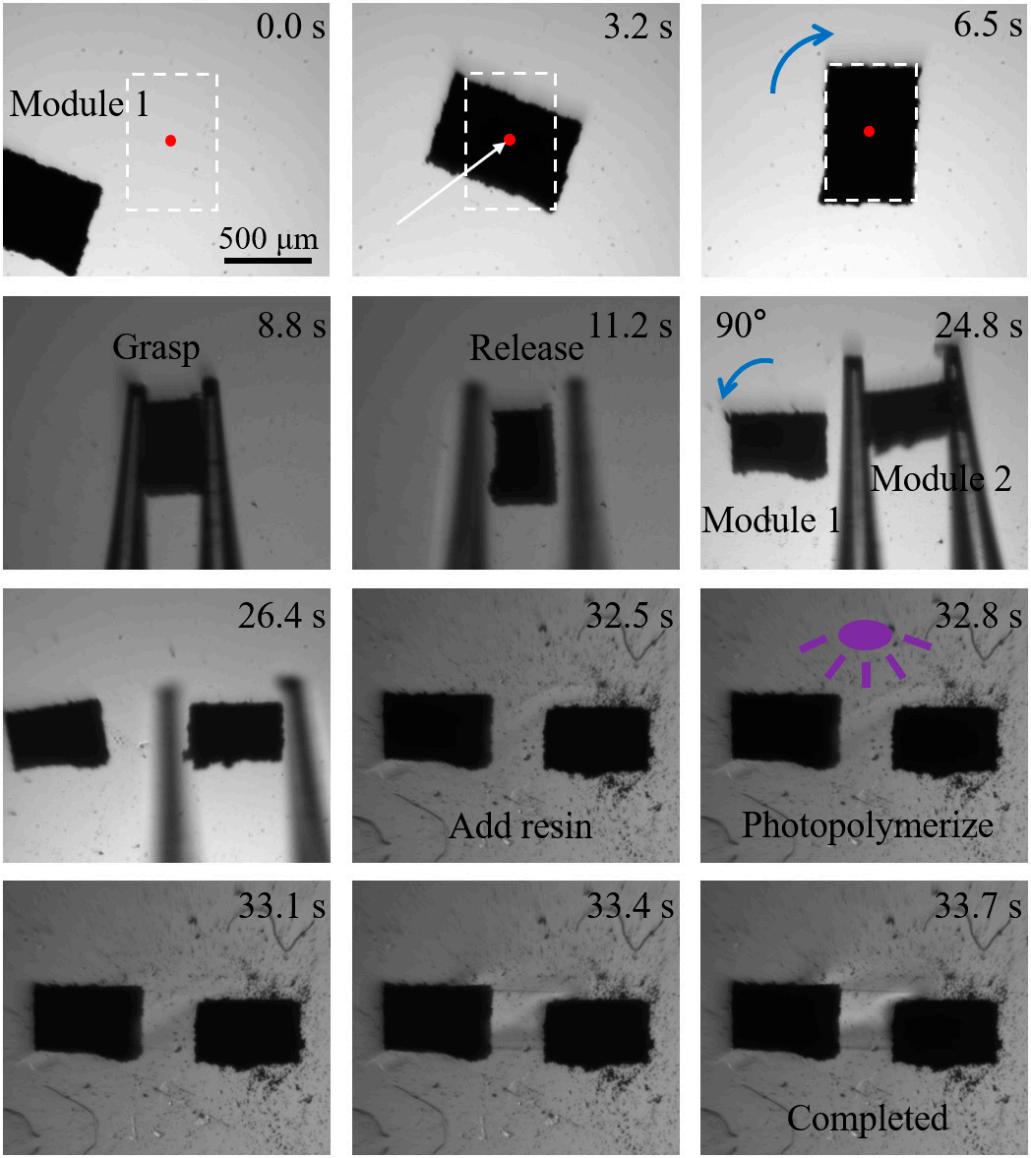
Fabrication of a simple soft 2-foot crawling microrobot.

Taking the fabrication of a simple soft 2-foot crawling microrobot as an example, the detailed fabrication process is shown in Fig. 3. We first found a pre-magnetized micro rectangular module at the corner of the visual field. The module was moved to the visual center immediately within 3.2 s. Then, the module was rotated for the following grasping. The module was then grasped by the 2-finger micro-hand and released in the assembling area. Positioning and rotating the first module took 11.2 s total. Then, the second module was found, grasped, and released at the desired position. Here, the previously placed module 1 was also rotated and positioned to realize the relative location of the 2 modules. The assembly in the second module took 15.2 s total. Then, the resin was added and we could see the change of the background. The 2 pre-magnetized micro rectangular modules were linked in the photopolymerized soft body. The photopolymerization took just 0.9 s. Finally, the simple soft 2-foot crawling microrobot was fabricated.

## Results

**Fig. 4.**
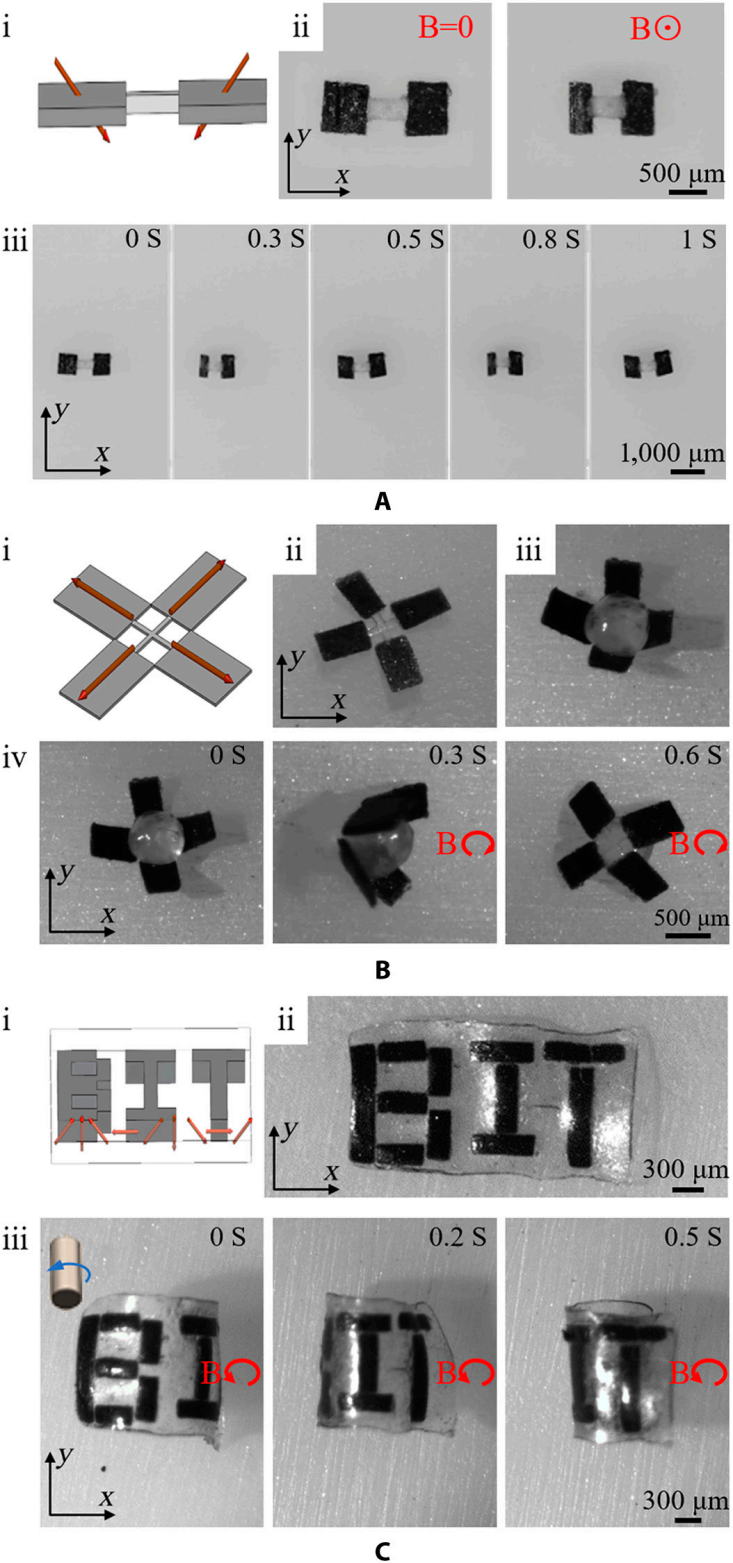
(A) Test of the soft 2-foot crawling microrobot. (B) Test of the claw-like robot. (C) Test of the rectangular microrobot with the “BIT” pattern.

Firstly, we tested the soft 2-foot crawling microrobot. The designed magnetization of the soft 2-foot crawling microrobot is shown in Fig. 4A(i). There are 2 locomotion states in one locomotion circle. The relaxed state with no magnetic field applied and the contracted state under the control of the magnetic field are shown in Fig. 4A(ii). The precise control of the 2-foot soft crawling microrobot in the *x*-*y* plane was achieved using the magnetic field generated by a permanent magnet. The 2-foot soft crawling robot bent to the maximum limit under the magnetic field with the strength of 60 mT in the positive *z* axis, and the microrobot switched to the relaxed state when the magnetic field was removed. Therefore, the robot could execute crawling locomotion by repeating the contraction and relaxation modes. The experimental results show that the robot crawls forward at a speed of 0.3 mm/s under the magnetic field with a frequency of 0.5 Hz as shown in Fig. 4A(iii).

Inspired by the art of origami, a claw-like robot is designed with 2 types of components: magnetic modules and a non-magnetic soft body. The magnetic modules were connected by the photopolymerized non-magnetic soft material (Fig. 4B(ii)). The magnetization profile of the robot is shown in Fig. 4B(i). The locomotion of the whole microrobot with no deformation could be actuated by a magnetic moment with low strength. The local deformation of the microrobot could only respond to the high-strength magnetic field, allowing for a sequential actuation of the microrobot via the magnetic system. When applying the magnetic field with a strength of 100 mT, every assembled magnetic module moved up and formed a cube-like shape for grasping. Figure 4B(iii) shows the grasp of a Polydimethylsiloxane (PDMS) microbead by deforming the microrobot into a semi-closed cube. Moreover, as shown in Fig. 4B(iv), the claw-like robot grasping a microbead can be rolled on the substrate under a rotating magnetic field with a frequency of 1 Hz.

We also designed a rectangular structure that can deform to a cylinder when applying an external magnetic field. The microrobot consisted of a photopolymerized soft rectangular body and the assembled magnetic modules according to the pattern “BIT”, which is the abbreviation of “Beijing Institute of Technology”. The designed magnetization profile is shown in Fig. 4C(i). As shown in Fig. 4C(ii), 3 letters B, I, and T assembled by pre-magnetized magnetic modules were packaged in a photopolymerized soft rectangular thin film. The rectangular structure with the letter pattern deformed into a cylindrical structure under a magnetic field with a strength of 100 mT. As shown in Fig. 4C(iii), the cylindrical robot rolled slowly on a substrate by applying a rotating magnetic field with a frequency of 0.2 Hz and displaying the B, I, and T pattern in turn.

## Discussion

In the proposed fabrication method of the magnetic soft microrobots, the micro modules as the building blocks could flexibly select various materials, include enough strong magnetic particles, and be pre-magnetized under a strong magnetic field to achieve designed local magnetic torque vectors. Photopolymerization was used to fabricate the soft body and package the micro pre-magnetized micro modules. The adopted micro-assembly brought the flexibility of material selection. More strong magnetic particles included in the micro modules could enable the fabricated microrobot to work under a low-strength magnetic field. The more effective pre-magnetization again enhanced this advantage.

The fabrication speed is mainly limited by the assembly process. The micro-hand driven by the piezo actuators can realize high-speed grasping and releasing, which greatly shorten the time utilized in the assembly process. Moreover, the fully automated assembly assisted by computer vision also improves the assembly efficiency obviously. The photopolymerization is conducted layer by layer. It holds the highest fabrication speed in fabricating 2D magnetic soft microrobots. The 3D magnetic soft microrobots with low requirements on the depth resolution could also be fabricated quickly.

The fabrication accuracy highly depended on the size of the magnetic modules, the operating accuracy, and the photopolymerization accuracy. The photopolymerization accuracy could be improved by the objective lens with high power and the DMD with high resolution. The other photopolymerization solution is also possible to be integrated with the proposed micro-hand assembly system. The modules could be minimized and magnetic particles with a size of several micrometers could also play as the magnetic modules, which enable the proposed fabrication method to fabricate microrobots under 100 μm. Compared with the traditional photopolymerization assembly systems that use magnetic fields to directly manipulate the micro magnetic modules, the proposed micro-assembly system has higher operating accuracy than the traditional photopolymerization assembly system. Taking operating a particle with the magnetic fields directly as an example, the average position error could be πr under the rolling mode, since the orientation is required. The orientation controlling accuracy could also be low if the magnetic field is applied to rotate the particles. The magnetic field strength of typical Helmholtz coils is not stable due to the increasing resistance caused by the heating effect. Thus, the accuracy of the magnetic vector is hard to guarantee. The proposed micro-assembly system can realize the precise control of the micro magnetic modules by micro-hand, which avoids the problem of low operation accuracy caused by the instability of magnetic field strength.

In this paper, we propose a new method based on photopolymerization and assembly for the fabrication of magnetic soft microrobots. Utilizing the micro-hand assembly system, magnetic modules with different shapes and materials are firstly arrayed with precise position and orientation control. Then, the developed photopolymerization system is employed to fix and link these modules with soft materials. Based on the proposed fabrication method, 3 kinds of soft magnetic microrobots were fabricated, and the fundamental locomotion was presented. The proposed fabrication method for the magnetic soft microrobots holds the advantages of fine control of the local magnetic vector, flexibility in selecting various materials, high magnetism of the fabricated magnetic soft microrobots, and acceptable fabrication efficiency. We believe that the fabrication strategy presented here could help accelerate the clinical application of magnetic microrobots.

## Data Availability

The data used to support the findings of this study are available from the corresponding author upon request.
